# Neonatal Severe Hyperparathyroidism: Anaesthetic Considerations for Removal of Pea-size Glands in Children

**DOI:** 10.4274/TJAR.2025.241409

**Published:** 2025-05-30

**Authors:** Uditi Parmar, Raylene Dias, Gayathri P, Madhuri Bamnote

**Affiliations:** 1 King Edward Memorial Hospital Seth Gordhandas Sunderdas Medical College Clinic of Paediatric Anaesthesiology, Mumbai, India

**Keywords:** Calcimimetics, hypercalcemia, neonatal severe hyperparathyroidism, parathyroidectomy

## Abstract

Neonatal severe hyperparathyroidism (NSHPT) is an extremely rare disorder with uncontrolled severe hypercalcemia and its clinical manifestations. It is caused by a mutation in the *calcium-sensing receptor (CaSR)* gene, which modulates the negative feedback of parathormone. We present anaesthetic management of two children with NSHPT who were posted for total parathyroidectomy as a life saving procedure. Both patients presented with lethargy, failure to thrive, and hypotonia. Intraoperative anaesthetic challenges include susceptibility to bradycardia, prolonged QT interval, precipitation of hypercalcemic crisis in the form of renal failure, hyperkalemia and electrocardiography changes, unpredictable response to neuromuscular blockade, susceptibility to recurrent laryngeal nerve injury, refractory hypocalcemia, which may start developing within six hours after surgery. Anaesthetic goals include preoperative optimisation of serum calcium with subcutaneous. Calcitonin, intravenous pamidronate and tablet cinacalcet, which are calcimimetics, maintenance of hydration and readiness to deal with intraoperative hypercalcemic crises. Anaesthetic management of NSHPT posted for total parathyroidectomy is challenging. To the best of our knowledge, there is no anaesthetic literature published to this day and only four surgical cases have been reported. Genome sequencing in both patients showed a *CaSR* gene mutation that is homozygous for a suspected pathogenic variant. Management requires a preoperative multidisciplinary approach for severe hypercalcemia and postoperative refractory hypocalcemia. These patients need lifelong calcium and vitamin D supplementation.

**Main Points**

• Neonatal severe hyperparathyroidism is an extremely rare disorder which is caused by inactivating mutations of calcium-sensing receptors present on the parathyroid gland.

• Parathyroidectomy is the definitive treatment.

• Pre-operative optimization of serum calcium is extremely challenging to prevent an intraoperative hypercalcemic crisis.

• Management requires preoperative multidisciplinary approach for severe hypercalcemia and postoperative refractory hypocalcemia. These patients need lifelong calcium and vitamin D supplementation.

## Introduction

Neonatal severe hyperparathyroidism (NSHPT) is an extremely rare disorder with prevalence of 2-5 cases per 100,000.^1, 2^ It is caused by inactivating mutations of calcium-sensing receptors (CaSR) present on parathyroid gland which modulates the inhibitory feedback of parathormone (PTH) release thus resulting in uncontrolled hyperparathyroidism with severe hypercalcemia.^3, 4^ Parathyroidectomy is a definitive treatment.^5^ We report anaesthetic management of two children with NSHPT, scheduled for total parathyroidectomy.

## Case Reports

### Case 1

**Figure 1:**
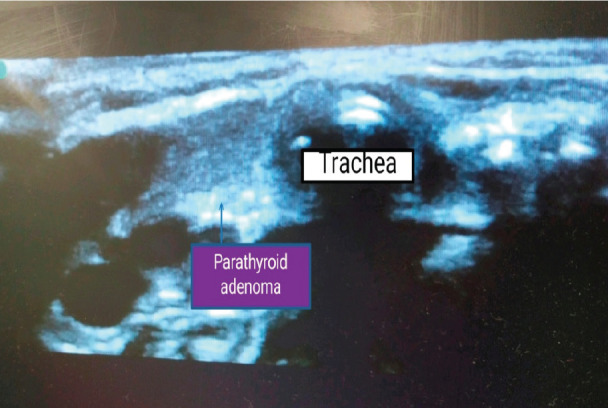
Ultrasound of neck showing parathyroid adenoma

**Figure 2:**
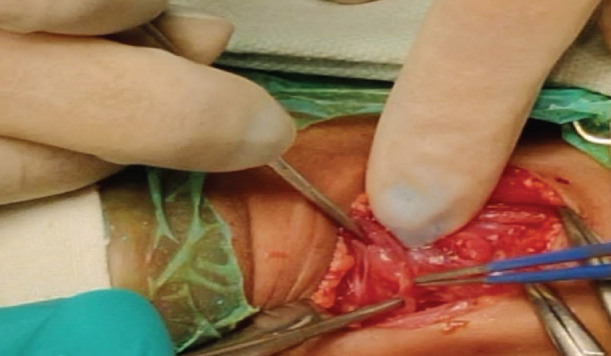
Right parathyroid lobule during surgical dissection

A 40-day-old term male infant weighing 2,840 kg was diagnosed with NSHPT on day-10 of life. The child presented with lethargy, excessive crying and failure to thrive. Subsequent investigations were done and revealed hypercalcemia [serum calcium of 18 mg dL^-1^, ionized calcium (iCa^2+^) 4.08 mmol L^-1^, serum PTH 971 pg mL^-1^ (range 15-65 pg mL^-1^)], and hyperkalemia (serum potassium 6.3 mEq L^-1^). The patient was treated in the neonatal intensive care unit (NICU) according to the hyperparathyroid protocol, which includes a first saline bolus (10 mL kg^-1^) followed by intravenous furosemide (1 mg kg^-1^), and then calcitonin 4 U kg^-1^ (every 12 hours) subcutaneously for the initial 48 hours. Hypercalcemia was still uncontrolled, hence three doses of intravenous pamidronate 1 mg kg^-1^ and tablet cinacalcet 0.5 mg kg^-1^ day-1 in three divided doses were given. Pre-operative electrocardiography (ECG) was suggestive of short QT interval. Ultrasonography neck revealed a parathyroid adenoma 4x2x4 mm in the right inferior parathyroid lobule with no displacement of the upper airway structures (Figure 1). The patient was planned for total parathyroidectomy due to uncontrolled hypercalcemia. On the day of surgery, the morning dose of cinacalcet was omitted as postoperative hypocalcemia was expected. Emergency drugs were kept ready, such as insulin for hyperkalemia, inhaled β2 agonists for hyperkalemia, and furosemide and calcitonin for hypercalcemic crisis. Intraoperative monitoring included non-invasive blood pressure (BP), pulse oximetry, ECG, temperature, and end tidal carbon dioxide (EtCO_2_). Baseline heart rate (HR) was 124 min^-1^ and BP 74/42 (54) mm Hg (50^th^-90^th^ centile). Near infrared spectroscopy (NIRS, INVOS^TM^ Somanetic Corp. Troy, MI) was monitored. Anaesthesia was induced with incremental doses of sevoflurane (2-6%), with oxygen and nitrous oxide (50/50). Intravenous fentanyl 2 µg kg^-1^ and intravenous cisatracurium 0.15 mg kg^-1^ were given, and the infant was intubated with a 3.5 mm uncuffed endotracheal tube. Anaesthesia was maintained with oxygen/nitrous oxide/sevoflurane minimum alveolar concentration (MAC-1-1.2). Under ultrasound guidance, the right radial artery was cannulated for intraoperative hemodynamic monitoring, blood gas, and serum PTH levels. Intraoperative train of four (TOF) monitoring was done for neuromuscular blockade. Plasmalyte was chosen as the intravenous fluid of choice as it is devoid of calcium, and was given at a maintenance rate of 10 mL kg^-1^ hr^-1^. Prior to surgical incision, 1 µg kg^-1^ fentanyl and intravenous Paracetamol 15 mg kg^-1^ were given. Intraoperatively, PTH levels were sent at the following time intervals i) Pre-resection (levels were 1,900 pg mL^-1^) ii) 20 minutes after removal of all four parathyroid glands (levels to 83 pg mL^-1^), this post resection drop in serum PTH along with pathological frozen sections confirmed successful resection of all four parathyroid glands (Figure 2). Post resection serum sodium was 134 mEq/L, potassium -5.6 mEq L and iCa^2+^>4 mmol L^-1^. Patient hemodynamics was stable throughout the surgical procedure without Inotropic support and NIRS values were within 20% of baseline (NIRS baseline values=75). At the end of the surgical procedure, the TOF count was >0.9 vocal cords were adducting and abducting well under C-MAC videolaryngoscope guidance following which trachea was extubated. Post extubation, the patient (post conceptional age >47 weeks) was shifted back to NICU for observation in view of risk of post-anaesthetic apnea. On postoperative day-1, serum PTH was 22 pg mL^-1^ serum calcium was 8.6 mg % and iCa^2+^ reduced to 1.93 mmol L^-1^ and patient was started on cap calcitriol 0.125 µg 12 hourly, vitamin D3 drops 2,000 IU day-1. The patient was discharged on postoperative day-13 with oral vitamin D3 and calcium supplementation.

### Case 2

A two year female child weighing 4.2 kg, with history of poor weight gain and delayed milestones was diagnosed as NSHPT on. Day-33 of life. The child received two doses of intravenous pamidronate for: high serum calcium 19.4 mg dL^-1^, iCa^2+^ 1.84 mmol L^-1^ and PTH 586 pg mL^-1^. Child was started on tab cinacalcet 7.5 mg. Preoperatively, serum calcium was 11.0 mg dL^-1^; iCa^2+^ 1.2 mmol L^-1^; and PTH 1,900 pg mL^-1^. Ultrasonographic evaluation of parathyroids showed two enlarged glands one at right midpole measuring 2*7 mm and in left lower pole measuring 2*4 mm. On the day of surgery, the morning dose of cinacalcet was omitted. The child was premedicated with intravenous midazolam 0.1 mg kg^-1^, and standard American Society of Anesthesiologists monitors, including non-invasive BP, pulse oximetry, ECG, and EtCO_2_, were attached. Baseline HR was 122 min^-1^ with BP 82/35 mmHg, NIRS values of 73 and ECG showed sinus rhythm. Child was induced with intravenous fentanyl 2 µg kg^-1^, intravenous propofol and intravenous atracurium 0.5 mg kg^-1^ and intubated with 3.5 mm microcuff® (AVANO, Roswell, GA) tube and maintained with oxygen/nitrous oxide and desflurane 4-6 (0.8 to 1.0 MAC). Intravenous paracetamol, 15 mg kg^-1^, was given as a part of multimodal analgesia. Ultrasound-guided vascular access was secured via the left brachiocephalic vein. All four parathyroids were excised and confirmed by frozen section and fall in PTH (Pre-resection PTH >1,900 pg mL^-1^, and 20 minutes post excision was 166 pg mL^-1^). Plasmalyte was given at the maintenance rate of 10 mL kg^-1^ h^-1^. Intraoperative hemodynamics were stable. Post-extubation, the patient was transferred back to intensive post-cardiac care unit for observation. On postoperative day-1, serum PTH was 11.2 pg mL^-1^, serum calcium was 8.8 mg dL^-1^, and iCa^2+^ was 1.13 mmol L^-1^. Patient was started on tab calcitriol 0.25 µg and was discharged on postoperative day-10 with calcium and vitamin D supplementation.

Genome sequencing in both patients showed *CaSR* gene mutation homozygous for suspected pathologic variant.

## Discussion

NSHPT requires emergency parathyroidectomy due to life-threatening manifestations like failure to thrive, anorexia, vomiting, irritability, lethargy, hypotonia, and seizures.^6, 7^ Bradycardia, short QT interval, and hypertension are other findings due to hypercalcemia. Long-term metabolic consequences include nephrocalcinosis that may cause permanent renal damage, skeletal muscle weakness, osteoporosis, and neurodevelopmental impairment. Our patients presented with hypercalcemia, hyperkalemia, lethargy and intolerance to feeding. Only four surgical cases have been reported so far. Calcitonin is given subcutaneously for the first 48 hours, as tachyphylaxis develops after that.^8^ Intravenous pamidronate (0.5-1 mg kg^-1^) inhibits macrophage and osteoclastic activity. Subsequent doses are repeated at 7-10 days interval. Cinacalcet is a calcimimetic drug and therefore increases the sensitivity of calcium receptors on parathyroid cells, thereby reducing PTH levels and thus decrease in serum calcium levels.^9, 10^ Pre-operative optimization of serum calcium is extremely challenging to prevent intraoperative hypercalcemic crisis.

Intraoperative anaesthetic challenges include susceptibility to bradycardia, prolonged QT interval, precipitation of hypercalcemic crisis in the form of renal failure, hyperkalemia and ECG changes, unpredictable response to neuromuscular blockade, and refractory hypocalcemia, which may start developing within six hours after surgery. Additional challenges include recurrent laryngeal nerve injury leading to vocal cord palsy, stridor, and tracheomalacia. Maintenance of hydration is important perioperatively to prevent hypercalcemic crisis. We used NIRS monitoring for cerebral oxygenation as a marker to rule out great vessel compression during neck dissection. Intraoperatively, the values were within 20% of baseline. TOF >0.9 was accepted for extubation and reversal. Neuromuscular blockade was antagonized using intravenous neostigmine 50 µg kg^-1^ and intravenous glycopyrrolate 10 µg kg^-1^ after generating adequate tidal volume and respiration with no signs of airway obstruction. In both cases, the intraoperative and postoperative course was uneventful.

## Conclusion

Anaesthetic management of NSHPT undergoing total parathyroidectomy is challenging. To the best of our knowledge, there is no anaesthetic literature published to this day. Management requires preoperative multidisciplinary approach for severe hypercalcemia and postoperative refractory hypocalcemia. These patients need lifelong calcium and vitamin D supplementation.
